# Disease profiles of detainees in the Canton of Vaud in Switzerland: gender and age differences in substance abuse, mental health and chronic health conditions

**DOI:** 10.1186/s12889-015-2211-6

**Published:** 2015-09-10

**Authors:** Karine Moschetti, Pierre Stadelmann, Tenzin Wangmo, Alberto Holly, Patrick Bodenmann, Jean-Blaise Wasserfallen, Bernice S. Elger, Bruno Gravier

**Affiliations:** Institut universitaire de médecine sociale et préventive, Unité d’evaluation des soins, University Hospital of Lausanne (CHUV), Route de la Corniche 10, 1010 Lausanne, Switzerland; Technology Assessment Unit (UET), University Hospital of Lausanne (CHUV), Lausanne, 1011 Switzerland; Institute for Biomedical Ethics, Institute for Biomedical Ethics, University of Basel, Bernoullistrasse 28, Basel, 4056 Switzerland; Institut d’économie et management de la santé (IEMS), HEC Lausanne and Département d’économétrie et d’économie politique (DEEP), University of Lausanne, Internef Building, Lausanne, 1015 Switzerland; Vulnerable Population Center, Department of Ambulatory Care and Community Medicine, University of Lausanne, Lausanne, 1011 Switzerland; Service of Correctional Medicine and Psychiatry (SMPP), University Hospital of Lausanne (CHUV), Prilly-Lausanne, 1008 Switzerland; Centre universitaire romand de médecine légale, University of Geneva, Geneva, Switzerland; IEMS - Plateforme interfacultaire en économie et management de la santé, University of Lausanne CH, Internef Building, Lausanne, 1015 Switzerland

## Abstract

**Background:**

Literature on the disease profile of prisoners that differentiates by age and gender remains sparse. This study aimed to describe the health of correctional inmates in terms of substance abuse problems and mental and somatic health conditions, and compare it by gender and age.

**Methods:**

This study examined cross-sectional data from the Canton of Vaud in Switzerland on the health conditions of detainees who were in prison on January 1, 2011 or entered prison in 2011. Health conditions validated by physician examination were reported using the International Classification of Diseases (ICD) version 10. The analyses were descriptive by groups of prisoners: the entire sample (All), Men, Older adults and Women.

**Results:**

A total of 1,664 individuals were included in the analysis. Men comprised 91.5 % of the sample and had a mean age of 33 years. The other 8.5 % were women and had an average age of 39. Older adults (i.e., age 50 and older) represented 7 % of the total sample. Overall, 80 % of inmates were non-Swiss citizens, but the proportion of Swiss prisoners was higher among the older adults (51 %) and women (29 %). Overall, 41 % of inmates self-reported substance abuse problems. Of those, 27 % were being treated by psychiatrists for behavioral disorders related to substance abuse. Chronic infectious diseases were found in 9 % of the prison population. In addition, 27 % of detainees suffered from serious mental health conditions. Gender and age had an influence on the disease profile of this sample: compared to the entire prison population, the older inmates were less likely to misuse illegal drugs and to suffer from communicable infections but exhibited more problems with alcohol and a higher burden of chronic health conditions. Female prisoners were more disposed to mental health problems (including drug abuse) and infectious diseases. In terms of chronic diseases, women suffered from the same conditions as men, but the diseases were more prevalent in women.

**Conclusion:**

It is important to understand the different disease profiles of prisoners by gender and age, as it helps identify the needs of different groups and tailor age-and gender-specific interventions.

## Background

The prison population is increasing worldwide [1], and it is widely accepted that prison inmates have poor health due to accumulated vulnerability factors such as traumatic life experiences, lack of a social network and low socioeconomic background [2]. Studies have shown that poly-morbidity among prisoners is high, with substance abuse, mental illnesses and infectious diseases as the most common health problems [3–5]. Substance abuse and dependence are common problems encountered among the prison population [6, 7]. It is not uncommon to observe 1 out of 3 or even 1 out of 2 inmates with drug abuse or dependence [8]. Smoking and alcohol problems are also common in prisons [9–11]. The widely-known negative health outcomes of tobacco use and secondhand smoke are highly problematic, and even more so in confined environments [12, 13].

Psychotic and personality disorders, including antisocial personality problems and major depression, are often identified among incarcerated people [14]. A recent literature review conducted in 24 countries revealed a constant prevalence of psychotic disorders over time [15]. Although they have conflicting figures, empirical studies on psychiatric and psychological disorders among young prisoners have confirmed the high prevalence of mental health problems [16].

With regard to prisoners’ physical health, there have been extensive investigations into infectious diseases such as human immunodeficiency virus and acquired immunodeficiency syndrome (HIV), hepatitis B and C and tuberculosis [5, 17]. Although there is still some uncertainty about the actual number of people living behind bars with these communicable illnesses, studies have consistently reported prevalence rates exceeding those observed in the community [18, 19]. In high income countries, the prevalence of HIV and tuberculosis has been found to be about 10 to15 times higher than in the civilian population [3, 20]. Moreover, the extent of infectious-disease transmission within prisons remains unknown; it is, however, not marginal [18, 21].

By comparison, non-infectious diseases have received less attention [19], and studies describing specific chronic conditions and their frequencies among offenders are limited. Available data highlight higher rates of diabetes, hypertension and asthma among inmates when compared to the general population in the USA [22], whereas in Switzerland the prevalence of these conditions among the prison population has been in line with that of the general population [3].

Most of the abovementioned studies present health information for young male prisoners, who form the bulk of the prison population (more than 90 % in most countries) [23]. Knowledge about the health status of special groups is limited. However, the number of older adults in prison is growing and is projected to increase further [24]. The growing older prisoner population is even viewed as a future health and economic crisis for both criminal justice systems and societies [25]. Today, in many countries, there is a tendency towards longer custodial sentences and sentences of undetermined length and/or life sentences, meaning that people entering prison when they are young are likely to remain when they are older [26]. Defining a strict threshold for when a prisoner becomes an “older prisoner” is complicated [27]. Prison contributes to accelerating the biological process of aging, and time spent in prison aggravates and increases medical problems [28]. Few papers in the literature tackle the health issues of this still small proportion of the prison population [26]. Reviews have suggested that prisoners aged 55 and older have a much higher prevalence of major diseases and functional impairments and multi-morbidity than younger prisoners [5] and non-prisoners of similar age [5, 26]. However, little is known about the extent to which chronic and degenerative diseases linked to aging – whose physiological signs (in the community) include hearing, visual and cognitive decline as well as reduced mobility –will increase the healthcare needs of older prisoners [29].

Women are another minority group in prison. They make up less than 8 % of the total prison population in Europe by country [23]. Like older prisoners, their population is reported to be growing. Explanations of this growth include a combination of social and political factors [30] linked to the nature of the offences committed. The majority of women are incarcerated for non-violent, property or drug-related crimes, for which they generally serve short sentences [31]. Like their male counterparts, or even more so, incarcerated women are disproportionately affected by multiple health problems such as substance abuse *(DAPHNE Projects data in MacDonald, 2013)*, HIV infection, sexually transmitted diseases and severe mental health problems [8, 32]. Furthermore, post-traumatic disorders, depression, self-harming behaviors and eating disorders are also common among female inmates [31, 32].

Limited research has been done on prisoners’ health and disease profile (including non-infectious, chronic diseases) that differentiates between these specific groups. In particular, there are very few studies in which the health problems among male, female and older prisoners have been estimated for the same prison setting/area and over the same time period. And yet, better knowledge of the epidemiological situation in prison – particularly the extent to which health is similar or different among male, female and older prisoners – is a prerequisite for evaluating prisoners’ healthcare needs and determining how to provide appropriate and targeted health services. This study examines exhaustive and recent health and demographic data on prisoners living in the Canton of Vaud, which is a region in the French-speaking part of Switzerland. The aim is to portray the health of correctional inmates, including their somatic and mental health conditions and substance abuse, and to differentiate by gender and age.

## Methods

In 2011, the prison population in Switzerland amounted to around 6,100 inmates distributed across111 institutions [33]. With about 80 prisoners per 100,000 inhabitants, Switzerland has one of the lowest incarceration rates in the world [34]. The present work reports data from the entire prison population of the Canton of Vaud. Approximately 1 in 10 permanent Swiss residents lives in the Canton of Vaud. It has one semi-open prison and 4 closed facilities for both men and women. The five institutions receive remand prisoners and prisoners serving sentences (see Table 1). The total number of stays in prison rose from 1,941 in 2002 to 2,610 in 2011.

**Table 1:** The four closed facilities in the Canton of Vaud

Prison’s name	Capacity	Type of sentences served	Gender of inmates
Bois Mermet	100	Custody on remand	Male
La Tuilière	80	Remand (men + women) and custodial sentences (women)	Male and female
La Croisée	170	Remand and short custodial sentences	Male
Plaine de l’Orbe	264	Long sentences	Male

The study used an exhaustive and unique dataset of demographics and medical information for all the prisoners in the closed facilities in 2011. It includes prisoners who were already in prison on January 1, 2011 and those who entered these facilities at any time throughout 2011. Cross-sectional data for the 2,610 inmate stays were individually collected by the Service of Correctional Medicine and Psychiatry (Service de Médecine et Psychiatrie Pénitentiaire–SMPP) affiliated with the University Hospital of Lausanne (CHUV). After data management, data cleaning and collapsing the multiple stays by the same individual over the 12 months into a single stay, the final sample consisted of 2,185 individuals. The study sample was then restricted to individuals who had a routine physician examination upon prison entry (*N* = 1,664). Under Swiss law, every incarcerated person should be seen by a general practitioner (GP) within 7 to 21 days following his/her entry [35, 36]. There may have been individuals who refused the entry examination. Also, time constraints such as a short stay or transfer to another prison may not have allowed for a meeting with a GP. Because the health information for these prisoners (*n* = 521) was incomplete, their data was removed from our analysis. All new prisoners receive a health evaluation by a nurse within 24 h of their entry in order to manage emergency situations and ensure continuity of care. In addition to the examinations by the GP and nurse, 44.8 % (*N* = 747) of the prisoners were examined by a psychiatrist and were found as having at least one psychiatric health conditions. The number of prisoners examined by a psychiatrist and without psychiatric diagnosis is negligible. Psychiatric examination happens at the request of health professionals (GP, nurses), penitentiary staff or the prisoners themselves. The psychiatric examination may also be a compulsory part of the prisoners’ sentence.

Thus, the information on inmates’ health conditions came from diagnoses made by a GP for somatic problems and by a psychiatrist for mental problems. These diagnoses relied on the prisoners’ case history, their self-reported information and additional medical tests when necessary. Health conditions were reported using the International Classification of Diseases (ICD) version 10, from which chronic diseases were selected and further examined. ICD-10 is the standard diagnostic tool for routine medical practice and epidemiology. It is used to monitor the incidence and prevalence of diseases and other health problems [37]. Moreover, using ICD-10 to report health conditions is imperative for psychiatry, since it is the only classification accepted for health- and disability-insurance purposes in Switzerland. We also used information from nurses’ consultations regarding inmates’ substance-related problems. In such cases, two complementary assessments for these disorders are available: that nurse’s assessment containing the inmate’s self-declarations, and the psychiatrist’s diagnosis.

Based on ICD-10, chronic conditions related to infections, skin problems, and the musculoskeletal, digestive, circulatory, endocrine, respiratory and nervous systems were sorted into groups. Additional subgroups were also created, including back pain, hypertension, diabetes, asthma, bronchitis, and migraines. For the purposes of our study, 4 prisoner groups were looked at: the entire study sample (All), men, older adults and women. Although the threshold for “older” prisoners varied from 50 to 65 years depending on the study [5, 27], we chose to define older adults as people aged 50 or older.

### Statistical analysis

The analyses were descriptive for each prisoner group: the entire sample (All), men, older adults and women. Statistical analyses included percentages, means, and standard deviations (SDs) or 95 % confidence intervals (CI). We used a Chi-squared test to explore associations between diseases and patients’ characteristics. Logistic regression models, including age and gender as adjustment variables, were run to test potential origin of related diseases. Statistical analyses were performed with STATA, the Data Analysis and Statistical software (version 12, StataCorp).

### Ethical considerations

The study was approved by the ethical commission of the Canton of Vaud, Switzerland (Protocol No. 388/12). Data were anonymized. As the study was retrospective and used only anonymized and aggregated data, individual agreement was not required.

## Results

### Socio-demographic characteristics

A total of 1,664 individuals examined by a physician upon entry were included in our analysis. The study sample was similar to the whole sample (*N* = 2,185) in terms of gender, age and nationality. The 521 individuals (24 %) who were excluded had stayed an average of 16 days, suggesting that they were released or transferred before the GP examination. Men comprised 91.5 % of the study sample, and their mean age was 32.8 years (SD 10.3, range 16–82 years). Women represented 8.5 % of the sample population and had an average age of 38.8 (SD 10.3, range 16–69 years). Older adults (aged 50+) made up 7 % of the sample (*n* = 136) and averaged 57.02 years old (SD (6.07, range 50–82 years). Among the older group, 90 % were men. Other demographic characteristics such as age group, marital status and nationality for the 3 study groups are provided in Table 2.

**Table 2:** Demographic characteristics, year of entry and length of prison stay for the prison population in the Canton of Vaud and in all of Switzerland for 2011

	Prisoners in the Canton of Vaud				National prison population (*) [55]	
	All	Men	Older adults	Women		Number of incarcerations
	(*N* = 1,664)	(*N* = 1,524)	(*N* = 136)	(*N* = 140)		
Gender in % (N)						
Male	91.5 (1524)	100 (1524)	89.70 (122)	0	92.0	7,988
Female	8.5 (140)	0	10.30 (14)	100	8.0	690
Age (mean, SD, range)	32.8 (10.3, [16, 82])	31.61 (10.6, [16, 82])	57.02 (6.07, [50, 82])	38.8 (10.3, [16, 69])	NA	
Age group as % of the total						
24 and under	23.38 (389)	24.28 (370)	0	13.59 (19)	25.79	2,212
25–34	39.60 (658)	39.83 (607)	0	36.42 (51)	37.35	3,241
35–44	22.77 (380)	21.85 (333)	0	33.57 (47)	21.99	1,908
Over 44	14.24 (237)	14.04 (214)	100	16.42 (23)	15.18	1,317
Nationality in % (N)						
Swiss	20.0 (332)	19.2 (292)	51.4 (70)	28.6 (40)	36.02	3,126
Non-Swiss	80.0 (1,332)	80.8 (1,232)	48.6 (66)	71.4 (100)	63.98	5,552
Eastern Europe	24.7 (411)	25.2 (385)	14.7 (20)	18.6 (26)	NA	
Africa	24.4 (406)	25.0 (381)	2.9 (4)	17.8 (25)	NA	
Western Europe	13.9 (231)	13.5 (206)	22.0 (30)	17.8 (25)	NA	
North Africa and Middle East	12.1 (202)	13.0 (197)	5.9 (8)	3.7 (3)	NA	
America	3.5 (58)	2.6 (40)	1.5 (2)	12.8 (18)	NA	
Asia	1.4 (24)	1.5 (23)	1.5 (2)	0.7 (1)	NA	
Marital status in % (N)§	(*N* = 1,641)	(*N* = 1,501)	(*N* = 135)	(*N* = 140)		
Divorced	13.5 (221)	12.2 (183)	42.9 (58)	27.1 (38)	NA	
Single	65.8 (1,080)	67.5 (1,014)	22.9 (31)	47.1 (66)	NA	
Married	19.2 (316)	19.2 (288)	28.1 (38)	20.0 (28)	NA	
Widowed	1.5 (24)	1.1 (16)	5.9 (8)	5.8 (8)	NA	
Year of entry into prison in % (N)						
Before 2010	10.7 (177)	10.4 (298)	28.0 (38)	23.6 (19)	NA	
In 2010	27.3 (454)	27.9 (425)	24.2 (33)	20.7 (29)	NA	
In 2011	62.0 (1,033)	61.7 (941)	47.8 (65)	65.7 (92)	NA	
Length of stay in 2011 in % (N)						
1 month	22.0 (366)	22.1 (338)	19.1 (26)	20.0 (28)	47.55	4,126
<3 months	54.8 (913)	55.0 (839)	47.0 (64)	52.8 (74)	67.72	5,877
< 6 months	73.1 (1,217)	72.7 (1,109)	62.5 (85)	77.1 (108)	79.47	6,896

(*) Characteristics of the national prison population refer to the number of incarcerations and not to the number of individuals

NA: Information not available

(§) This information is missing for 23 observations

From the sample population, 80 % of inmates were non-Swiss citizens, but the proportion of Swiss detainees was higher among the older adults (51 %) and women (29 %). Nationalities of 99 different countries were reported, with the main regions being Eastern Europe and Africa for the full sample, Western Europe for the older adults, and Africa and Eastern Europe for the women (Table 2).

Eleven percent of all prisoners had entered prison prior to 2010 (28 % of the older adults and 23 % of the women). Most of the prison population entered prison during the study year (62 %). Among them, 22 % had stays of less than one month, and 73 % of less than 6 months.

Comparing the characteristics of our sample with the prison population of Switzerland for the same year (see Table 2), our study sample showed the same proportion of men and women and a similar age distribution, but the Swiss nationality was less represented. There were also differences in the length of stays: in 2011, stays lasting less than one month amounted to 22 % in our sample compared with more than double that nationally. On the other hand, 27 % of detainees in the Canton of Vaud had a stay longer than 6 months, versus 20 % nationally. The proportion of prisoners in the sample serving long sentences was higher than in the national prison population. This may be related to the presence of facilities for long custodial sentences in the Canton of Vaud that are not necessarily available in all Swiss prisons.

### Disease profile of the prisoners (All)

Prisoners suffered from multiple health problems, as reported in Tables 3 and 4. Overall, 41 % of inmates self-declared substance-abuse problems, such as with illegal drugs, pharmaceuticals or alcohol. Of that group, 27 % were being treated by psychiatrists for behavioral disorders related to psychoactive substance use (Table 3). Psychiatric examination also revealed evidence of psychiatric problems other than substance abuse. The prevalence of personality and behavioral disorders among inmates was 16.2 %, and 15.9 % for neurotic disorders. Prisoners also suffered from schizophrenia, mood disorders and behavioral syndromes. Overall, 35.1 % suffered from some form of mental disorder other than substance use.

**Table 3:** Mental health conditions and dependencies for the four groups

Proportion of prisoners with a mental illness (ICD 10 codes) and psychiatric follow-up	All		Men		Older adults		Women		Chi 2	
	(*N* = 1664)		(*N* = 1524)		(*N* = 136)		(*N* = 140)		*p*-value	
	%	95 % CI	%	95 % CI	%	95 % CI	%	95 % CI	Sex	Age
Mental illness classified as a behavioral disorder due to psychoactive substance use (F10-F19)	26.3	24.2-28.4	25.6	23.4-27.9	19.1	12.8-26.7	32.8	25.1-41.3	NS	0.048
Drugs (F11-12-13-19)	18.0	16.1-19.9	17.2	15.4-19.2	3.7	1.2-8.4	25.7	18.7-33.8	0.013	<0.001
Pharmaceuticals (F13)	5.2	4.2-6.4	5.3	4.2-6.6	2.2	0.4-6.3	4.2	1.6-9.0	NS	NS
Alcohol (F10)	9.5	8.0-11.1	9.4	8.0-11.0	16.2	10.4-23.4	10.7	6.1-17.0	NS	0.006
Schizophrenia (F 20-F29)	5.3	4.3-6.5	5.1	4.0-6.3	6.6	3.0-12.2	7.1	3.5-12.8	NS	NS
Mood [affective] disorders (F30-F39)	2.2	1.6-3.1	1.7	1.1-2.4	3.7	1.2-8.4	7.8	4.0-13.6	<0.001	NS
Neurotic, stress-related and somatoform disorders (F40-F49) = mainly reaction to severe stress, and adjustment disorders	15.9	14.1-17.8	14.8	13.0-16.7	13.2	8.0-20.1	27.1	19.9-35.3	<0.001	NS
Behavioral syndromes associated with physiological disturbances and physical factors (F50-F59)	1.9	1.3-2.7	1.9	1.3-2.7	0.0	0.0	1.4	0.2-5.0	NS	NS
Disorders of adult personality and behavior (F60-69)	16.2	14.5-18.0	15.1	13.3-16.9	25.7	18.6-34.0	27.8	20.6-36.0	<0.001	<0.002
Mental retardation (F70-79) and other disorders (F80-99)	2.8	2.0-3.6	2.5	1.8-3.5	3.7	1.2-8.4	5.0	2.0-10.0	NS	NS
Any mental disorder (excluding substance use)	35.1	32.8-37.5	33.3	31.0-35.7	42.6	34.2-51.4	55.0	46.3-63.4	<0.001	0.050

NS: not significant (at a 5 % level)

**Table 4:** Main chronic health conditions of the 4 groups

Proportion of prisoners with diseases (ICD 10 codes)	All		Men		Older adults		Women		Chi 2		Prevalence data in the general Swiss population
	(*N* = 1664)		(*N* = 1524)		(*N* = 136)		(*N* = 140)		*P*-value		
	%	95 % CI	%	95 % CI	%	95 % CI	%	95 % CI	Sex	Age	%
Infectious diseases	8.9	7.6-10.4	8.1	6.8-9.6	4.4	1.6-9.3	17.1	11.3-24.4	<0.001	NS	
HIV (B20-B24-Z21)	2.7	1.9-3.6	2.1	1.1-2.9	1.5	0.1-5.2	9.0	5.0-15.3	<0.001	NS	0.3 [56]
Hepatitis B (B16 B18)	1.5	1.0-2.2	1.6	1.0-2.3	0.0	0-2.6	0.7	0.01-3.9	NS	NS	0.3 [57]
Hepatitis C (B17 B18.2)	5.0	4.1-6.2	4.6	3.6-5.6	2.9	0.8-7.3	10.7	6.1-17.0	0.002	NS	0.7-1 [57]
Tuberculosis (A15 A16)	0.36.	0.13-0.78	0.4	0.08-0.7	0.0		0.0		NS	NS	0.007 [56]
Tuberculosis sequelae (B90)	0.66	0.33-1.17	0.7	0.3-1.1	0.0		0.0				
Diseases of the musculoskeletal system (M10 M15 M54 M54.4)	12.8	11.2-14.5	12.8	11.1-14.5	21.3	14.7-29.2	12.8	7.8-19.5	NS	0.002	
Back pain only (M54 M54.4)	9.0	7.8-10-6	9.0	7.6-10.6	14.7	9.2-21.8	10.7	6.1-17.0	NS	0.020	8.8 for 34 < age < 44; 11.9 for 55 < age < 64 [58]
Diseases of the digestive system (K21 K25-K 26 K29 K58 K70)	7.6	6.4-9.0	7.6	6.4-9.1	11.7	6.9-18.4	7.1	3.4-12.7	NS	NS	
Diseases of the circulatory system (I05-I09 I10 I20 I25 I50 I87.2)	6.5	5.4-7.8	6.3	5.2-7.7	33.0	25.2-41.6	7.8	4.0-13.62	NS	<0.001	
Hypertension (I10)	5.5	4.3-6.6	5.3	4.2-6.5	25.7	18.6-33.9	6.4	2.9-11.8	NS	<0.001	2.2-8.1 for age < 49; 23.6 for 50 < age < 64 [59]
Skin problems (B36.0 D10-D36 L20-30 L40 L50 L70)	8.1	6.8-9.5	8.3	6.9-9.8	5.8	2.5-11.2	5.7	2.5-11	NS	NS	
Endocrine diseases (E66 E10-14 E78)	5.1	4.1-6.2	5.2	4.1-6.4	22.8	16.0-30.7	4.3	15.9-9.1	NS	<0.001	
Diabetes (E10-14)	2.1	1.5-2.9	2.1	1.5-3.0	13.23	8.0-20.1	2.1	0.4-6.1	NS	<0.001	0.6-1.8 for age < 49; 5.8 for 50 < age < 64 [59]
Diseases of the respiratory system (J30 J32 J41 J45)	5.5	4.4-6.7	5.4	4.3-6.6	9.5	5.2-15.8	6.4	3-11.9	NS	0.029	
Asthma (J45)	2.3	1.7-3.2	2.1	1.4-2.9	2.9	0.8-7.3	5.0	2.0-10.0	0.030	NS	2.5 for age 30-39 yrs [60]
Bronchitis (J41)	2.1	1.5-2.9	2.2	1.5-3.1	5.9	2.5-11.2	1.4	0.1-5.0	NS	0.002	
Diseases of the nervous system (G40 G43.0)	1.4	0.9-2.0	1.1	0.6-1.8	2.2	0.4-6.3	4.2	1.5-9	0.002	NS	
Migraines (G43.0)	0.7	0.3-1.2	0.4	0.1-0.8	0.7	0.1-4.0	4.2	1.5-9.0	<0.001	NS	7.7 for age < 24-9.3 for age < 44 [58]
Self-reported subs. misuse or dependence											
Drug misuse	32.2	29.6-34.5	32.0	29.6-34.3	5.9	2.5-11.2	35.0	27.1-43.5	NS	<0.001	Cannabis: men 12; women 4 15 < age < 34 [61]
Pharmaceutical misuse	10.8	9.3-12.3	10.2	8.7-11.7	4.0	1.6-9.3	16.0	10.7-23.6	0.024	0.013	
Alcohol misuse	18.9	17.0-20.8	19.0	16.9-20.9	24.2	17.3-32.5	17.8	11.9-25.2	NS	NS	
Cigarette misuse	46.4	43.9-48.8	46.9	44.4-49.4	38.9	30.7-47.7	40.0	31.8-48.6	NS		28.2 % in 2012 [59]; 32.4 for men; 24.2 for women;
Any drug, pharmaceutical, or alcohol misuse	40.8	38.4-43.1	40.7	38.2-43.2	29.4	21.9-37.8	42.1	33.8-50.7	NS	0.005	

NS: not significant (at a 5 % level)

Chronic infectious diseases were found in 9 % of the prison population. Hepatitis C and HIV were the most common. In terms of somatic chronic health problems, the most frequently identified diseases were those affecting the musculoskeletal system (13 %). Other problems are detailed in Table 4. In addition, almost 1 prisoner in 2 self-reported smoking. Among all prisoners, 40 % had at least one somatic chronic health condition.

### Older adults

A lower rate of mental illness related to psychoactive substance use and confirmed by a psychiatrist was found among older inmates compared to prisoners under 50 (19 % vs. 27 %). Notably, compared to prisoners under 50, older adults were significantly less likely to misuse illegal drugs (*p* < 0.0001) but more likely to abuse alcohol (*p* < 0.0001). Self-reported information on substance use was 30 %–45 % higher than what was confirmed by psychiatric follow-up. In all, 3.5 % of the older prisoners had the 3 dependencies.

Data indicated that 42.6 % of the older inmates suffered from at least one psychiatric disorder other than substance use. Twenty-six percent suffered from personality disorders, 13 % from neurosis, 6.6 % from schizophrenia and 4 % from mood disorders.

Of the older adults, 1.5 % were found to have HIV and 3 % to have hepatitis C. There was no significant difference compared to prisoners under 50 (*p* = 0.35 and *p* = 0.23).

Diseases of the musculoskeletal, circulatory and respiratory systems were more frequent in the group of older adults than in the full sample. The association between being an older adult and suffering from a health condition was statistically significant for back pain (*p* = 0.02), hypertension (*p* < 0.0001), bronchitis (*p* = 0.002) and endocrine disorders (*p* < 0.0001) and almost statistically significant for digestive problems (*p* = 0.058). Overall, 60 % of older prisoners had at least one chronic health condition (excluding mental problems).

### Women

Female inmates had a higher prevalence of psychiatric problems than the men. Behavioral disorders related to psychoactive substance use were confirmed by a specialist to exist in one-third of the female detainees, significantly higher than in the men. Female inmates were more often found to have drug addictions (26 % vs. 17.2 %, *p* = 0.013). However, no gender differences were evident for misuse of pharmaceuticals (4.2 % for women *vs.* 5.3 % for men) or alcohol (10.7 % for women *vs* 9.4 % for men). In examining the self-declared substance abuse assessments, pharmaceutical misuse was significantly more frequently reported by women than men (16 % vs. 10 %; *p* = 0.024). A large proportion (40 %) of female inmates reported smoking.

Regarding other mental disorders, results showed that female prisoners were more disposed to mental illnesses than men, with the increased prevalence ranging from 25 % to 75 %. The only exception was behavioral syndromes, for which the rate was slightly lower than for men (1.5 % vs. 2 %). Moreover, there was a significant gender difference for 3 groups of mental disorders: mood disorders, “reaction to severe stress” and personality disorders (see Table 3).

Among the various infectious diseases, HIV and hepatitis C infections were significantly more frequent among female prisoners than male prisoners (9.2 vs. 2.7 % *p* < 0.0001 for HIV and 10 % vs. 5.2 % *p* = 0.002 for hepatitis C). However, less than 1 % had hepatitis B (no gender difference found). Back pain, hypertension, asthma and migraines were more frequent among female than male inmates. While back pain and hypertension didn’t show a significant gender difference, asthma and migraines did (5 % vs. 2.3 % *p* =0. 03; resp. 4.2 % vs. 0.7 % *p* < 0.0001).

### Chronic diseases related to origin in the study sample

Compared to people from Western Europe (including Swiss people), prisoners from Africa and Eastern Europe were less likely to suffer from substance abuse (OR 0.11 95 % CI 0.07-0.16 and OR 0.27 95 % CI 0.19-0.37), but those from the Middle East were more likely (OR 1.71 95 % CI 1.23-2.39), particularly with pharmaceutical (OR 7.70 95 % CI 4.50-13.17) and alcohol (OR 2.04 95 % CI 1.32-3.15) dependencies. Lower prevalence rates for alcohol misuse were found in Eastern European (OR 0.41 95 % CI 0.25-0.69) and African inmates (OR 0.30 95 % CI0.16-0.54) compared with Western European prisoners. Detainees from the Middle East had a higher burden of digestive disorders (OR 2.20, 95 % CI 1.24-3.89), prisoners from Africa more frequently reported hypertension (OR 2.43, 95 % CI 1.22-4.85) than prisoners from Western Europe, but less frequently endocrine disorders (OR 0.41 95 % CI 0.16-1.04; *p* = 0.062).

## Discussion

Using a cross-sectional, large-scale and comprehensive dataset on prisoner health, this study portrayed the epidemiological situation of the prison population in the Canton of Vaud, Switzerland. First, unlike other studies that use prison population samples, this study analyzed exhaustive data on prisoners in Vaud Canton over the same time period. Second, while many studies tend to emphasize either male prisoners or young prisoners, we differentiated the disease profiles between groups of inmates (All, Men, Older adults and Women). Third, this study provides direct comparisons between the health of two minority inmate groups (older adults and women) and the younger and male prisoners on four aspects of health: substance abuse, mental health, infectious diseases and chronic conditions. As such, it contributes to the literature on the health burden among prisoners in general, and that of older and female prisoners in particular. Finally, this study underlines the fact that prisoners are suffering from multiple morbidities combining psychiatric and somatic disorders. Beyond these findings, our study also made some specific and important points, which are highlighted below.

From the literature, it is evident that prisoners are at higher risk for drug problems than those living in the community [5, 6]. There is a notable heterogeneity among studies, and estimated prevalence of drug abuse varies from 10 % to 53 % in male offenders. In a Swiss context, active use of illegal drugs was found in 40 % of remand prisoners [3]. In our study, the two available measures on drug problems revealed differences: 18 % of prisoners were diagnosed with drug abuse problems in the psychiatric follow-up, but in the entry examination conducted by nurses, 32 % of inmates self-declared being active drug users. The difference can be explained by 3 factors. The main explanation is that many detainees declared themselves to be drug abusers even when not addicted and without necessarily having or showing symptoms requiring a psychiatrist examination. If such prisoners do not explicitly seek psychiatric help to treat their drug misuse problem, they are not seen by psychiatrists. Also, the estimated rate from self-reports may be overestimated. Some prisoners may report problems for the purpose of obtaining medications/treatments that would help them bear incarceration [21]. Finally, the organizational constraints of incarceration may imply that some prisoners do not have time or opportunity to see a psychiatrist during their stay. The number of prisoners with a psychiatric diagnosis of substance use may thus be underestimated, especially for men, given their greater numbers. The heterogeneity of data on substance abuse in prison underscores the challenge of obtaining an accurate prevalence of drug use/misuse in the prison population [38], especially when some prisoners hide their dependencies to avoid sanctions [21].

Our findings indicate that the need for substance abuse treatment is great and important for inmates. It is also now recognized that systematically rehabilitating prisoners with substance abuse problems often remains an empty hope [39]. Previous therapy consisting of providing drug-free programs have now been replaced by substitution –methadone-maintenance programs –at least in some European countries [40]. This has been the therapeutic approach adopted in the prisons included in this study for several years.

Communicable diseases, such as hepatitis B and C and HIV were found at rates between 5 and 10 times greater than those reported in the Swiss general population (Table 3). Our results are consistent with those in France and Canada [20, 41, 42] and those found in the large remand Swiss prison in Geneva [3]. With a rate of tuberculosis among the studied inmates >50 times that of Swiss civilians, our findings confirm the public health issues related to this disease. As pointed out by the WHO (Stop TB Strategy [43]), the prison population is one of the risk groups that needs special attention. Efforts already implemented to improve detention conditions (including overcrowding, hygiene conditions and nutrition) should be further developed to better contain the transmission of this disease. This is particularly relevant in a population made up of a disproportionate number of individuals with high risk factors for tuberculosis (HIV, intravenous drug use, low socioeconomic background as well as geographical origin [43]).

The prevalence of common conditions such as back pain, hypertension, diabetes and asthma among prisoners was in the same range as that observed in their counterparts living outside the prison in the country (Table 4). Compared to recent US studies, prisoners from the Canton of Vaud tended to show lower rates of hypertension, asthma and diabetes [8]. Moreover, compared to a publication reporting health problems among detainees in Geneva [3], our results exhibited lower rates of skin problems, migraines and asthma, but higher rates of back pain, diabetes and hypertension. There are several explanations to account for these differences. First, our study sample was older, which may explain the higher rate of hypertension and diabetes. Second, instead of focusing on remand prisoners, our sample included inmates not only on remand awaiting trial, but also on trial and serving short and long sentences. Prisoners serving sentences have to work in prison under sometimes sub-optimal or not very ergonomic conditions, leading to health problems such as back pain. Third and importantly, the methodology differed in that the health conditions of prisoners were reported according to 2 different international classifications: International Classification of Primary Care versus ICD-10.

Compared to the entire study population, the older adult prisoners showed a different disease profile. First, older prisoners did not suffer from the same type of addictions. Drug abuse was significantly less frequent among prisoners aged 50+, whereas alcohol abuse disorders were more frequent. This finding is in line with results from Scottish prisoners where the group aged 40–64 showed a higher proportion of prisoners with alcohol dependence than the group aged 25–39 [9]. Additionally, a recent analysis of the health profile of older prisoners in north-west England revealed that the prevalence of alcohol abuse was higher than that of drug abuse [26]. A second feature of the disease profile of the older adults was the higher prevalence of mental disorders compared to their younger counterparts. Excluding the dependence disorders, about 40 % of older prisoners had mental health problems. In our study, schizophrenia was reported at 6.5 % and personality disorders at 26 %, while the English study found rates of 5 % and 20 % respectively [26]. Third, communicable infections were significantly less frequent among older prisoners than younger prisoners. Finally, older prisoners showed a higher prevalence of more common chronic diseases than their younger counterparts, and than older people living outside the prison. Overall, these findings confirmed the previous results indicating that a large proportion of older prisoners suffer from psychiatric illnesses and chronic health problems [44, 45]. The differences in disease profiles between younger and older prisoners raise questions about the required healthcare needs of prisoners, not only now but also in the future.

In prison, healthcare needs linked to aging have emerged as great challenges. Addressing the delivery of healthcare services to older prisoners with multiple morbidities as well as age-related degenerative diseases raises inescapable issues with respect to the equivalence of care between prison and the community [46]. Provision of equivalent care takes on an extra dimension with the management of cognitive impairments such as dementia [47] and transitional end-of-life care needs [48]. Another issue that impacts the health of older prisoners is the structural environment of prison facilities. The prisons were initially designed for vigorous and independent people, and were not built to provide decent care to people who might need assistance with basic personal everyday life tasks and/or who may have geriatric syndromes.

Gender also had an influence on the disease profile among the study population. We found that substance abuse was more often encountered in female than in male prisoners. Our results reinforced previous findings showing gender differences in the prevalence of drug use disorders among prisoners [6, 8]. In Europe, previous studies have found problematic drug use ranging from 10 % to 50 % in female prisoners [49]. Up-to-date results based on DAPHNE Projects data and reported by McDonald [30] revealed that 75 % of female prisoners in Germany, and 98 % in Scotland, had addiction problems with drugs, alcohol, prescription drugs and eating disorders. We also found that female prisoners were more likely than men to have co-existing psychiatric disorders such as personality disorders, depression and anxiety. This is in line with results reported in the literature [50, 51] showing that women were also more likely to self-harm and commit suicide than male inmates [32]. In addition, although it was not examined in our study due to a lack of available data, many female prisoners might also be mothers or have worries about children. As a result, the mental health needs of female prisoners are complex.

Stress and anxiety associated with incarceration may be compounded by the overcrowded conditions and violence existing in prison facilities. If overcrowding significantly worsens the physical health of prisoners by encouraging the spread of communicable disease, it seems likely that overcrowding could also adversely impact the mental health of prisoners [52]. Mental illness also complicates treatment for co-morbidities, such as HIV, which requires strict adherence to drug therapy and careful monitoring for adverse drug interactions [53]. Addressing the mental health needs of male and female prisoners will improve not only their quality of life but also that of the prison staff and those without mental health illnesses [54].

Compared to men, women in prison had a higher burden of communicable diseases (almost double), especially for HIV and hepatitis C. This is similar to estimations from French prisons [20]. As for common chronic diseases, the incarcerated women in the study region suffered from the same health conditions as men but the diseases were more prevalent in women. Additionally, female prisoners have specific needs related to reproductive health (menstruation, menopause, pregnancy), and such needs are often more challenging than those of men [30, 51]. The growing number of incarcerated women raises concerns about gender-specific issues and needs in places initially designed for men.

### Limitations and strengths

The aim of this study was to describe the health status of prisoners by investigating substance abuse and mental health and chronic health problems, including infectious diseases. The study provides much needed knowledge on health in prison and helps to identify prisoners’ specific healthcare needs in general as well as by age and gender. However, our decision to focus on chronic conditions does not allow us to provide a complete picture of health problems since daily illnesses and symptoms (injuries) were excluded. Special focus on these more acute disorders would be interesting. Errors in collecting and reporting data may exist even if several checks were carried out to ensure coherence. Having restricted the analysis to people who were examined upon entry limited the underestimation errors of not only infectious and communicable diseases, but also chronic diseases. Indeed, the examination upon entry as a routine provides a detailed medical history of the prisoners and as such may identify symptoms leading to a precise diagnosis. Although examination on entry might also help identify other problems that individuals arrive with and that are associated with life experience, they may be more difficult to categorize. Concerning whether the data can be generalized, we analyzed the characteristics of those prisoners who did not receive a physician entry visit and were removed from our results. We found that they were similar to those included in the study in terms of gender, nationality and marital status. The excluded group was slightly younger (mean age of 30 years, SD = 9.5). Their average length of stay was 16 days. We have concluded that excluding these individuals has very little impact on the data and does not prevent the results from being generalized. Finally, it cannot be excluded that the prevalence of psychiatric disorders might be slightly underestimated since not all prisoners had the opportunity to have a psychiatric examination. This is because their stay was short or because they were transferred to another prison in Switzerland (or they were deported to their country of origin). However, this was a very small proportion of the prisoners since specific training for both nurses and penitentiary staff allows them to identify psychiatric needs efficiently. They are therefore able to set up an examination relatively quickly. More than 95 % of new prisoners are seen by nurses, assuring good medical follow-up/management. Thus the percentage of inmates in need of a psychiatric examination who do not get the opportunity is very small indeed and has little impact on the data.

Strengths of this study include the large sample size and use of recent, exhaustive data from a prison population. This population includes different types of inmates, ranging from prisoners on remand awaiting trial to prisoners serving a sentence. Thus it closely mirrors the prison population of all of Switzerland, thereby increasing its external validity and allowing for extrapolation of our results.

## Conclusion

This paper contributes to the knowledge on disease profiles of incarcerated persons with a particular focus on two minority groups– older inmates and female inmates– on which the literature remains sparse. The specificities of their health situations help identify priorities for group-specific interventions. There is an urgent need for research priorities and policy initiatives on “aging and health” in the criminal justice system. Devising them will be beneficial both for the inmates and prison health professionals, who may feel powerless without the specific knowledge to deal with this group. Also, it seems crucial to evaluate the extent to which the aging prison population will be taxing on societies in terms of financial and other resources. Programs already in place that appropriately target female inmates should be sustained and, where possible, reinforced. More longitudinal research investigating health trends over time and health prior to and during detention are also needed. Such work would allow us to determine the changes that occur in the health profile of prisoners upon incarceration as well as the extent to which health of prisoners deteriorates or improves in prison, and the factors associated with those health changes.

## Abbreviations

ICD: International Classification of Disease
GP: General practitioner
CI: Confidence interval
OR: Odds ratio
SD: Standard deviation
